# Supplementary material for: Flexibility in motor timing constrains the topology and dynamics of pattern generator circuits
Source: Nat Commun. 2018 Mar 6;9:977. doi: 10.1038/s41467-018-03261-5 (PMC5840308; doi:10.1038/s41467-018-03261-5)
Supplement: Supplementary file 1 — Supplementary Information [file 41467_2018_3261_MOESM1_ESM.pdf]

Supplementary Information

Cengiz Pehlevan, Farhan Ali, Bence Ölveczky

Supplementary Note 1: Flexible modifications to temporal patterns by changes in synaptic weights

Here we present the general conditions under which a pattern generator network can implement specific and independent modifications to the temporal structure of its output by changes to its synaptic weights.

We consider a pattern generator network with N neurons. Let W_{ij} be the strength of synaptic connections from the jth to the ith neuron. The network produces K temporal intervals, the durations of which are given by $I^{(\alpha)}$, with $\alpha = 1, \dots, K$. These intervals are functions of the synaptic connectivity matrix, $\mathbf{W}$, a fact which we denote by the notation $I^{(\alpha)}(\mathbf{W})$.

Specific trajectories in synaptic weight space

Specificity of temporal modifications requires the existence of trajectories in synaptic weight space along which only a single interval changes. Suppose an interval β is to be modified specifically. Is there a trajectory in synaptic weight space along which only interval β changes?

We use the notation $\mathbf{W}^{(\beta)}(l)$ to denote such a specific trajectory, where l parametrizes the trajectory. We choose our parametrization such that $l = 0$ is the original network's synaptic weight matrix. We assume that $\mathbf{W}^{(\beta)}(l)$ varies smoothly with l, requiring at least differentiability, and assume that along these trajectories no new synapses are formed, i.e. synapses with zero weight remain with weight zero. We require $I^{(\beta)}\left(\mathbf{W}^{(\beta)}(l)\right)$ to be a strictly increasing function of l, and assume that it varies smoothly along the trajectory, again at least differentiably (see also below). Then, for specificity, along $\mathbf{W}^{(\beta)}(l)$, the following conditions must be satisfied:

$$I^{(\alpha)}\left(\mathbf{W}^{(\beta)}(l)\right) = I^{(\alpha)}\left(\mathbf{W}^{(\beta)}(0)\right) \quad \text{if } \alpha \neq \beta,$$
$$I^{(\alpha)}\left(\mathbf{W}^{(\beta)}(l)\right) \text{ is an arbitrary, strictly increasing function of } l. \tag{SI.1}$$

The temporal range, that is the maximum and minimum values of $I^{(\alpha)}\left(\mathbf{W}^{(\beta)}(l)\right)$, for which (SI.1) must hold depends on the needs of the organism and the behavior that the pattern generator is governing. Complemented with such a range, (SI.1) is the most general statement for the specifity requirement.

What can be learned from such a statement? For ease of notation, we switch to a vector representation of the matrix $\mathbf{W}^{(\beta)}(l)$, where $\mathbf{w}_{+}^{(\beta)}(l)$ refers to a vector composed from the non-

zero elements of $\mathbf{W}^{(\beta)}(l)$. Then, taking a derivative of both sides of and applying the chain rule, one gets:

$$\delta_{\alpha\beta}\frac{dI^{(\beta)}\left(\mathbf{w}_+^{(\beta)}(l)\right)}{dl} = \left.\frac{\partial I^{(\alpha)}(\mathbf{w}_+)}{\partial \mathbf{w}_+}\right|_{\mathbf{w}_+=\mathbf{w}_+^{(\beta)}(l)} \cdot \frac{d\mathbf{w}_+^{(\beta)}(l)}{dl}, \qquad \text{(SI. 2)}$$

where $\delta_{\alpha\beta}$ is the Kronecker delta. The left-hand sides of these equations are all zero except when $\alpha = \beta$. Eq. (SI.2) implies that along a specific trajectory, gradients of non-target interval durations with respect to synaptic weights must be orthogonal to the tangent vector of the trajectory.

To solve Eq. (SI.2), we first get rid of $I^{(\beta)}\left(\mathbf{w}_+^{(\beta)}(l)\right)$ dependence by a reparametrization. Let us remember that $I^{(\beta)}\left(\mathbf{w}_+^{(\beta)}(l)\right)$ is an arbitrary and strictly increasing function of l. For any choice of $I^{(\beta)}\left(\mathbf{w}_+^{(\beta)}(l)\right)$, we define

$$\bar{l}(l) \coloneqq I^{(\beta)}\left(\mathbf{w}_+^{(\beta)}(l)\right) - I^{(\beta)}\left(\mathbf{w}_+^{(\beta)}(0)\right). \qquad \text{(SI. 3)}$$

In terms of this new parameter, (SI.2) becomes,

$$\left.\frac{\partial I^{(\alpha)}(\mathbf{w}_+)}{\partial \mathbf{w}_+}\right|_{\mathbf{w}_+=\mathbf{w}_+^{(\beta)}(\bar{l})} \cdot \frac{d\mathbf{w}_+^{(\beta)}(\bar{l})}{d\bar{l}} = \delta_{\alpha\beta}. \qquad \text{(SI. 4)}$$

Once a solution to this equation is found, other solutions that trace the same trajectory with different speeds can be constructed by reparametrizations that respect the strictly increasing nature of $I^{(\beta)}\left(\mathbf{w}_+^{(\beta)}(l)\right)$.

With initial conditions $\mathbf{W}^{(\beta)}(0) = \mathbf{W}$, Eq. (SI.4) defines a non-linear system of K ordinary differential equations (one for each α) for S unknowns, S being the number of non-zero elements of $\mathbf{W}$. In ordinary situations $K << S$ and hence these equations will typically be underdetermined with infinitely many solutions. However, caution must be exercised to avoid trajectories, $\mathbf{w}_+^{(\beta)}(\bar{l})$, crossing pathological points such as:

1. When $I^{(\alpha)}(\mathbf{w}_+)$ is not differentiable. This could happen, for example, if the network is poised at a bifurcation point. We exclude such cases, because such networks will not exhibit the robustness required of a pattern generator circuit.

2. When $\frac{\partial I^{(\beta)}(\mathbf{w}_+)}{\partial \mathbf{w}_+} = \mathbf{0}$. We exclude a vanishing gradient because it would prevent the pattern generator to implement local changes to the βth interval and hence is not flexible.

To be able to say whether the conditions listed above, and possible others, seriously restrict the existence of specific trajectories, one needs know the details of the particular temporal pattern generator network. The models we present in the main text allow for sufficiently large variations

of synaptic weight matrices around their baseline value for at least $\approx 10\%$ changes in interval durations, as can be seen in our simulations, and hence should exhibit specific trajectories. The argument we presented can be separately repeated for all the intervals that the pattern generator produces.

Until now, we have only considered specific changes to intervals starting from the original network connectivity. What if a specific change to another interval is required after a specific change to the network has already been made? Such a scenario can be generalized to task-specific combinations of intervals and interval changes, each of which would add constraints of the form Eq. (SI.4), starting from different network connectivities. In this paper, we only focus on changes from the original network connectivity.

Independent trajectories in synaptic weight space

Next, we discuss independent trajectories. These are trajectories along which multiple intervals can change simultaneously without interference, by which we mean that their rates of change do not suffer compared to how they would change on their own. Suppose $\beta_1, \dots, \beta_r$ are the intervals targeted for change. Is there an independent trajectory in synaptic weight space, $\mathbf{W}^{(\beta_1,\dots,\beta_r)}(l)$, for changing the duration of these intervals?

To quantify interference, we need to compare the rate of interval changes along $\mathbf{W}^{(\beta_1,\dots,\beta_r)}(l)$ to the rate of interval changes along specific trajectories. Therefore, to have a well-defined problem we need to assume the existence of specific trajectories (see above). Then, we can state the condition for independence as:

$$I^{\alpha}\big(\mathbf{W}^{(\beta_1,\dots,\beta_r)}(l)\,\big) = I^{\alpha}\big(\mathbf{W}^{(\beta_1)}(l)\,\big) + \cdots + I^{\alpha}\big(\mathbf{W}^{(\beta_r)}(l)\,\big), \qquad \text{(SI.5)}$$

where $I^{\alpha}\big(\mathbf{W}^{(\beta_i)}(l)\,\big)$ are specific trajectories. The parametrization of specific trajectories can be chosen freely for the current discussion, but in a biological setting that will be discussed in the next section, l relates to the learning rate during single- and multiple-target learning. Taking derivatives with respect to l and applying the chain rule on both sides gives:

$$\left.\frac{\partial I^{(\alpha)}(\mathbf{w}_+)}{\partial \mathbf{w}_+}\right|_{\mathbf{w}_+=\mathbf{w}_+^{(\beta_1,\dots,\beta_r)}(l)} \cdot \frac{d\mathbf{w}_+^{(\beta_1,\dots,\beta_r)}(l)}{dl} = \sum_{i=1}^{r} \left.\frac{\partial I^{(\alpha)}(\mathbf{w}_+)}{\partial \mathbf{w}_+}\right|_{\mathbf{w}_+=\mathbf{w}_+^{(\beta_i)}(l)} \cdot \frac{d\mathbf{w}_+^{(\beta_i)}(l)}{dl}. \quad \text{(SI. 6)}$$

Note that the terms in the summation on the right-hand side are zero except when $\alpha = \beta_i$, due to specificity. Hence, independent paths must be orthogonal to gradients of non-target intervals. For each $\beta_1, \dots, \beta_r$ combination, this amounts to K equations, one for each interval α, for S unknowns. In ordinary situations, $K << S$ and hence these equations will typically be underdetermined and solutions to them can be found, provided that the dependence of interval durations on synaptic weights are not pathological, as discussed above, for the required range of interval durations. This argument can be separately repeated for all possible independent interval combinations.

Once a set of specific paths are found locally (meaning around $l = 0$), a linear combination of specific paths gives an independent path. Formally,

$$\mathbf{w}_+^{(\beta_1,\dots,\beta_r)}(\epsilon) = \mathbf{w} + \epsilon \sum_{i=1}^{r} \left. \frac{d\mathbf{w}_+^{(\beta_i)}(l)}{dl} \right|_{l=0} + O(\epsilon^2), \tag{SI.7}$$

where ϵ is a small parameter, solves (SI.6) to order ϵ^2. There could, however, exist other independent paths.

The existence of specific and independent trajectories in synaptic weight space is a minimum and necessary requirement for flexible time-keeping, but such paths must be found using biologically plausible learning rules, and we discuss them next.

Biologically plausible reinforcement learning of specific and independent temporal changes

Until now we discussed the existence of trajectories in weight space along which modification to individual intervals is specific and independent. In reality, such trajectories have to be traced by synaptic plasticity during trial-and-error learning. The information available at each synapse is very restricted: a signal about the produced target interval as well as knowledge about the synaptic weight of the synapse and the states of the pre- and postsynaptic neurons. This poses a serious challenge for how to implement specific and independent modifications to timing in biologically plausible networks. What neurons as a population can do, instead of solving (SI.2) and (SI.6), is to learn to increase reward. In fact, there are many biologically plausible reinforcement learning models in the literature[1–3] which suggest plasticity rules that find the direction of maximum reward increase, i.e. the 'gradient', in synaptic weight space when averaged across many trials.

Leaving the trial-and-error aspect of learning aside (it is addressed in the main paper), let us for a moment assume that the network has calculated a trajectory that is a gradient ascent on reward, $R^{(\beta)} = R^{(\beta)}(I^{(\beta)})$ (β is the target interval):

$$\frac{d\mathbf{w}_+^{(\beta)}}{dl} = \eta \frac{\partial R^{(\beta)}}{\partial \mathbf{w}_+} = \eta \frac{dR^{(\beta)}}{dI^{(\beta)}} \frac{\partial I^{(\beta)}}{\partial \mathbf{w}_+}, \tag{SI.8}$$

where η is a positive parameter that governs the rate of change. Then, using (SI.2) and (SI.8), we deduce that biologically plausible specific modification of interval duration requires orthogonality:

$$\left. \frac{dR^{(\beta)}}{dI^{(\beta)}} \frac{\partial I^{(\beta)}}{\partial \mathbf{w}_+} \cdot \frac{\partial I^{(\alpha)}}{\partial \mathbf{w}_+} \right|_{\mathbf{w}_+ = \mathbf{w}_+^{(\beta)}(l)} = \frac{dR^{(\beta)}}{dI^{(\beta)}} M_{\beta\alpha} = D_{\beta\alpha}(l), \tag{SI.9}$$

where $\mathbf{D}(l)$ is a diagonal matrix with positive diagonals, $\mathbf{M}$ is the interference matrix defined in the main text, and $\mathbf{w}_+^{(\beta)}(l)$ is now the trajectory obtained by the reinforcement learning algorithm. Note that this relation has to hold at each point along the trajectory. Equation (SI.9) is

a design constraint on a pattern generator network and in the main text we check whether the right-hand side is indeed a diagonal function for various possible pattern generator networks (Figs. 2E, 3D and 5C).

Simultaneous and independent modifications to multiple intervals require multiple reinforcement signals delivered to the network. Here we assume that the combined effect of such reinforcement is given by an addition of each reinforcement. Borrowing notation from the previous section:

$$R^{(\beta_1,\dots,\beta_r)} = R^{(\beta_1)} + \cdots + R^{(\beta_r)}. \tag{SI.10}$$

With this assumption, specificity in a reinforcement learning experiment would imply independence, as, locally, independent paths will be linear combinations of specific paths, as in (SI.7).

Supplementary Note 2: Flexible time-keeping in feedforward networks requires a one-to-one mapping between synapses and the interval durations they affect

Here we discuss flexible time-keeping in a pattern generator network with feedforward architecture.

In this section, we make explicit references to "time points", $t^{(\alpha)}$, which mark the beginnings and ends of intervals, i.e. $I^{(\alpha)} = t^{(\alpha)} - t^{(\alpha-1)}$. We label layers by Greek symbols. We make the following general assumptions:

1. The mapping between network activity and time is layer specific: the αth time point, $t^{(\alpha)}$, is a function of only the activity of the neurons in the αth layer.

2. As also implicit in the previous assumption, time increases with progression through the feedforward network. Activity in each layer codes for the start of an interval and the end of the previous one.

3. Only the initial layer, which we call 0th layer, receives external input.

For example, the first spike time of the neurons in α^{th} interval may code for the beginning of the αth interval and the end of the $(\alpha-1)^{\text{th}}$ interval.

The case of a single neuron per layer

For simplicity, let's first assume a single neuron per layer. We will discuss the generalization of our results to multiple neurons per layer in the next section.

Because in the case of a single neuron per layer, there is a one-to-one match between intervals and synaptic weights in a sense that will be described below, we change our notation for synaptic weights and neuron labeling slightly. Neurons and the layers they belong to are labeled with Greek subscripts, as opposed to the Latin subscripts of the previous sections. In addition, the synaptic weight between the neuron in $(\alpha-1)^{\text{th}}$ layer and α^{th} layer is denoted by W_α.

We denote the activity of the αth neuron by $r_\alpha(t)$, which can be spikes or firing rate. The feedforward architecture makes $r_\alpha(t)$ a function of the activity of the presynaptic neuron $r_{\alpha-1}(t)$ and the connection between them W_α:

$$r_\alpha(t) = r_\alpha\big(W_\alpha, r_{\alpha-1}(t)\big). \qquad \text{(SI.11)}$$

Interval boundaries, on the other hand, depend on the activity of neurons

$$t^{(\alpha)} = t^{(\alpha)}(r_\alpha(t)) = t^{(\alpha)}(W_\alpha, r_{\alpha-1}(t)). \qquad \text{(SI.12)}$$

Claim 1: In a feedforward network with a single neuron per layer, under the assumptions listed above, flexible time-keeping requires that the αth interval, $I^{(\alpha)} = t^{(\alpha)} - t^{(\alpha-1)}$, depends only on W_α, i.e.

$$\frac{\partial I^{(\alpha)}}{\partial W_\beta} = 0 \qquad \text{if } \alpha \neq \beta,$$
$$\frac{\partial I^{(\alpha)}}{\partial W_\beta} \neq 0 \qquad \text{if } \alpha = \beta. \qquad \text{(SI.13)}$$

Hence, synaptic weights affect only specific interval durations. Moreover, Eq. (SI.13) is satisfied if and only if

$$\frac{\partial t^{(\alpha)}}{\partial W_\beta} = \begin{cases} 0, & \text{if } \beta > \alpha \\ \dfrac{\partial t^{(\alpha)}}{\partial W_\alpha} & \text{if } \beta \leq \alpha \end{cases} \quad \text{and} \quad \frac{\partial t^{(\alpha)}}{\partial W_\alpha} \neq 0. \qquad \text{(SI. 14)}$$

Hence, flexibility requires the synaptic weight to shift all upstream time points by the same amount.

Proof. First, we prove (SI.14) implies (SI.13) and vice versa. Then we prove (SI.13).

1. That (SI.14) implies (SI.13) follows from the application of (SI.14) to the definition of interval durations, i.e. $I^{(\alpha)} = t^{(\alpha)} - t^{(\alpha-1)}$.

   To prove that (SI.13) implies (SI.14), first note that,

   $$\frac{\partial t^{(\alpha)}}{\partial W_\beta} = 0, \qquad \beta > \alpha, \qquad \text{(SI. 15)}$$

   always holds, since the activity of postsynaptic neurons do not affect the activity of presynaptic neurons in a chain. Next, assume (SI.13). For some β, consider all $\alpha > \beta$, for which $\frac{\partial I^{(\alpha)}}{\partial W_\beta} = 0$. By $I^{(\alpha)} = t^{(\alpha)} - t^{(\alpha-1)}$, this implies that $\frac{\partial t^{(\alpha)}}{\partial W_\beta} = \frac{\partial t^{(\alpha-1)}}{\partial W_\beta} = \cdots = \frac{\partial t^{(\beta)}}{\partial W_\beta}$, where the last equality comes from the case $\alpha = \beta + 1$. If $\alpha = \beta$, $\frac{\partial I^{(\alpha)}}{\partial W_\alpha} = \frac{\partial t^{(\alpha)}}{\partial W_\alpha} \neq 0$, by (SI.15) and (SI.13).

2. We will prove (SI.13) using strong induction.

   We prove the claim for the first interval as the base case. Note that by (SI.15),

   $$\frac{\partial I^{(1)}}{\partial W_\beta} = 0, \qquad \beta > 1. \qquad \text{(SI. 16)}$$

   Flexible learning requires the learning matrix to be diagonal with positive diagonal elements (see (SI.19)). The first row and column of the matrix are given by:

$$M_{1,\alpha} = M_{\alpha,1} = \sum_{\beta=1}^{K} \frac{\partial I^{(1)}}{\partial W_\beta} \frac{\partial I^{(\alpha)}}{\partial W_\beta} = \frac{\partial I^{(1)}}{\partial W_1} \frac{\partial I^{(\alpha)}}{\partial W_1} \propto \delta_{\alpha 1}. \quad \text{(SI.17)}$$

Hence, flexible learning requires

$$\frac{\partial I^{(\alpha)}}{\partial W_1} \propto \delta_{\alpha 1}. \quad \text{(SI.18)}$$

Now the (strong) induction step: Assume that the claim holds for interval durations, $I^{(\alpha)}$, $\alpha = 1$ to $\alpha = \gamma - 1$. We prove that this implies that the claim holds for $\alpha = \gamma$. Flexible learning requires interference matrix to be diagonal with positive diagonal elements. The γth row and column of the matrix are given by:

$$M_{\gamma,\rho} = M_{\gamma,\rho} = \sum_{\beta=1}^{K} \frac{\partial I^{(\gamma)}}{\partial W_\beta} \frac{\partial I^{(\rho)}}{\partial W_\beta} \propto \delta_{\gamma\rho}. \quad \text{(SI.19)}$$

Looking at the right hand side, we note that the terms in the summation for which $\beta > \min(\gamma, \rho)$ are 0 by (SI.15), and for which $\beta < \gamma$ are 0 by our induction assumption. Then, the only possibly non-zero term in the summation is:

$$M_{\gamma,\rho} = M_{\rho,\gamma} = \frac{\partial I^{(\gamma)}}{\partial W_\rho} \frac{\partial I^{(\rho)}}{\partial W_\rho} \propto \delta_{\gamma\rho}. \quad \text{(SI.20)}$$

Since flexible learning requires interference matrix to be diagonal with positive diagonal elements.

$$\frac{\partial I^{(\gamma)}}{\partial W_\beta} \propto \delta_{\gamma\beta}. \quad \text{(SI.21)}$$

□

Next, we present a sufficiency condition for a feedforward network to exhibit flexible timing.

Claim 2: Suppose all neurons' activities are time-invariant with respect to their pre-synaptic partners, i.e. if the pre-synaptic neuron activity is shifted in time by t', the result is a temporal shift in the post-synaptic neuron's activity by t'. Then, a feedforward network exhibits flexible time-keeping if changing a weight W_α leads to a time-shift only of the αth neuron's activity.

Proof: If perturbing W_α leads to a time-shift in the αth neuron's activity, all later neuron activities will be time shifted by the same amount by time-invariance property, and therefore no other interval than $I^{(\alpha)}$ will be affected.

□

The case of multiple neurons per layer

The argument presented above applied to having a single neuron per layer. The argument can be extended to multiple neurons. We chose a notation that makes the layered structure of the feedforward network explicit. Let's denote by $r_{\alpha,i}(t)$ the activity of the ith neuron in the αth layer and let $W_{\alpha,ij}$ be the synaptic weight from the jth neuron in the $(\alpha-1)^{\text{th}}$ layer to the ith neuron in the αth layer. We assume that there are N_α neurons in each layer. The feedforward architecture makes $r_{\alpha,i}(t)$ a function of the activity of the presynaptic neurons $r_{\alpha-1,j}(t)$ and the synapses between them $W_{\alpha,ij}$:

$$r_{\alpha,i}(t) = r_{\alpha,i}\big(W_{\alpha,i1},\dots,W_{\alpha,iN_{\alpha-1}},r_{\alpha-1,1}(t),\dots,r_{\alpha-1,N_{\alpha-1}}(t)\big). \tag{SI.22}$$

Interval boundaries, on the other hand, depend on the activity of neurons

$$\begin{aligned} t^{(\alpha)} &= t^{(\alpha)}\left(r_{\alpha,1}(t),\dots,r_{\alpha,N_\alpha}(t)\right) \\ &= t^{(\alpha)}\left(W_{\alpha,i1},\dots,W_{\alpha,iN_{\alpha-1}},r_{\alpha-1,1}(t),\dots,r_{\alpha-1,N_{\alpha-1}}(t)\right). \end{aligned} \tag{SI.23}$$

Claim 3: In a feedforward network with a multiple neurons per layer, under the assumptions listed above, it is sufficient for flexible time-keeping that the αth interval, $I^{(\alpha)} = t^{(\alpha)} - t^{(\alpha-1)}$ depend only on $W_{\alpha,ij}$, i.e.

$$\begin{aligned} &\frac{\partial I^{(\alpha)}}{\partial W_{\beta,ij}} = 0 \qquad \text{if } \alpha \neq \beta, \\ &\frac{\partial I^{(\alpha)}}{\partial W_{\beta,ij}} \neq 0 \qquad \text{if } \alpha = \beta \text{ for at least one } \{i,j\} \text{ pair} \end{aligned} \tag{SI.24}$$

If we further have the extra condition that intervals cannot increase with weight increases, i.e. $\frac{\partial I^{(\alpha)}}{\partial W_{\beta,ij}} \leq 0$, than (SI.24) is both necessary and sufficient for flexible time-keeping.

Proof. We will prove this claim using strong induction. We note that the claim already holds for $\beta > \alpha$ as activity of postsynaptic neurons do not affect activity of presynaptic neurons in a chain,

$$\frac{\partial I^\alpha}{\partial W_{\beta,ij}} = 0, \qquad \beta > \alpha. \tag{SI.25}$$

We prove the claim for the first interval as the base case. Flexible learning requires interference matrix to be diagonal with positive diagonal elements (see (SI.9)). The first row and column of the matrix are given by:

$$M_{1,\alpha} = M_{\alpha,1} = \sum_{\beta=1}^{K}\sum_{i=1}^{N_\beta}\sum_{j=1}^{N_{\beta-1}} \frac{\partial I^{(1)}}{\partial W_{\beta,ij}}\frac{\partial I^{(\alpha)}}{\partial W_{\beta,ij}} = \sum_{i=1}^{N_\beta}\sum_{j=1}^{N_{\beta-1}} \frac{\partial I^{(1)}}{\partial W_{1,ij}}\frac{\partial I^{(\alpha)}}{\partial W_{1,ij}}. \tag{SI.26}$$

If (SI.24) holds, then

$$M_{1,\alpha} = M_{\alpha,1} \propto \delta_{\alpha 1}, \tag{SI.27}$$

ensuring flexible learning. Further, if intervals cannot increase with weight increases, then (SI.24) for $\alpha = 1$ is necessary for flexible learning.

Now the (strong) induction step: Assume that the Claim 3 holds for $\alpha = 1$ to $\alpha = \gamma - 1$. We prove that this implies that the claim holds for $\alpha = \gamma$. Flexible learning requires interference matrix to be diagonal with positive diagonal elements. The γth row and column of the matrix are given by:

$$M_{\gamma,\rho} = M_{\rho,\gamma} = \sum_{\beta=1}^{K} \sum_{i=1}^{N_\beta} \sum_{j=1}^{N_{\beta-1}} \frac{\partial I^{(\gamma)}}{\partial W_{\beta,ij}} \frac{\partial I^{(\rho)}}{\partial W_{\beta,ij}}. \tag{SI.28}$$

Looking at the right hand side, we note that the terms in the summation for which $\beta > \min(\gamma, \rho)$ are 0 by (SI.25) and for which $\beta < \gamma$ are 0 by our induction assumption. Then, the only possibly non-zero terms in the summation are:

$$M_{\gamma,\rho} = M_{\rho,\gamma} = \sum_{i=1}^{N_\beta} \sum_{j=1}^{N_{\beta-1}} \frac{\partial I^{(\gamma)}}{\partial W_{\gamma,ij}} \frac{\partial I^{(\rho)}}{\partial W_{\gamma,ij}}. \tag{SI.29}$$

If (SI.24) holds, then

$$M_{\gamma,\rho} = M_{\rho,\gamma} \propto \delta_{\gamma\rho}, \tag{SI.30}$$

ensuring flexible learning. Further, if intervals cannot increase with weight increases, then (SI.24) for $\alpha = \gamma$ is necessary for flexible learning.

□

Supplementary Note 3: Flexibility range of a chain of integrate-and-fire neurons

Here we present a calculation of the range of synaptic weight strengths within which a chain of integrate-and-fire neurons exhibit timing flexibility. Integrate-and-fire neurons are arranged in a chain with a single neuron per layer. We will work in the synaptic weight regime where the activity propagates in the chain with each neuron producing a single spike, which mark interval boundaries. The sub-threshold dynamics of neuron α's membrane potential is given by:

$$\tau \frac{dV_\alpha}{dt} = -(V_\alpha - V_{rest}) + W_\alpha E\big(t - t^{(\alpha-1)}\big). \qquad \text{(SI. 31)}$$

Here, $E(t)$ is the excitatory post-synaptic potential (EPSP), which is 0 for $t < 0$ and normalized to unit area, $\int_0^\infty d\,t\,E(t) = 1$. $t^{(\alpha-1)}$ is the spike time of the $(\alpha-1)^{\text{th}}$ neuron. When the neuron reaches threshold, V_{th}, the neuron produces a spike and the membrane potential is reset to V_R. We assume that the neuron is at rest potential, V_{rest}, when the first pre-synaptic spike arrives. The membrane potential of the neuron after the first pre-synaptic spike and before its own first spike is given by:

$$V_\alpha(t + t^{(\alpha-1)}) = V_{rest} + W_\alpha \int_0^t \frac{dt'}{\tau} e^{-(t-t')/\tau} E(t'). \qquad \text{(SI. 32)}$$

The minimum synaptic weight strength for producing a spike happens when the maximum value of the membrane potential hits the spiking threshold. Taking a derivative of the membrane potential and setting it to zero, we get an implicit equation for the time of the maximum potential, $t_{\max}$:

$$E(t_{\max}) = \int_0^{t_{\max}} \frac{dt'}{\tau} e^{-\frac{(t_{\max}-t')}{\tau}} E(t'). \qquad \text{(SI. 33)}$$

Then, the minimum synaptic weight at which a spike is produced is given by:

$$W_{\alpha,\min} = \frac{V_{th} - V_{rest}}{\int_0^{t_{\max}} \frac{dt'}{\tau} e^{-(t_{\max}-t')/\tau} E(t')}. \qquad \text{(SI. 34)}$$

As an example, assume that $E(t) = \Theta(t)\, e^{-t/\tau_s}/\tau_s$, where $\Theta(t)$ is the step function and τ_s is the synaptic time constant. When $\tau_s \neq \tau$, $t_{\max} = \frac{\tau\tau_s \ln(\tau/\tau_s)}{\tau-\tau_s}$ and $W_{\alpha,\min} = (V_{th} - V_{rest})\tau_s \left(\frac{\tau_s}{\tau}\right)^{\frac{\tau}{\tau_s-\tau}}$. When $\tau_s = \tau$, $t_{\max} = \tau$ and $W_{\alpha,\min} = (V_{th} - V_{rest})\tau e$. $W_{\alpha,\min}$ monotonically increases with τ_s, starting with $W_{\alpha,\min} = 0$ for $\tau_s = 0$, which is the delta-function EPSP limit. The intuition behind this behavior is that as τ_s get bigger, less of the EPSP falls in the time window of integration ($\sim \tau$) and the rise in membrane potential becomes smaller. To compensate for this effect, the synaptic weight has to get stronger.

The maximum synaptic weight strength for flexible timing depends on the configuration of intervals in the network. One can increase the synaptic weight until a second postsynaptic spike is generated without any effect on the durations of downstream intervals. The second spike, however, increases the excitation in the $(\alpha+1)^{\text{th}}$ neuron, and may lead to earlier spiking of the $(\alpha+1)^{\text{th}}$ neuron. Therefore, a lower bound for the maximum synaptic weight strength for flexibility, is the strength at which a second spike is generated by the αth neuron. The membrane potential after the first spike (but before a possible second spike) is

$$V_\alpha\left(t+t^{(\alpha)}\right)=V_{rest}+W_\alpha\int_{t^{(\alpha)}-t^{(\alpha-1)}}^{t}\frac{dt'}{\tau}e^{-\frac{(t-t')}{\tau}}E(t'). \qquad \text{(SI.35)}$$

Note that $t^{(\alpha)}$ is a function of W_α as synaptic weight strength affects the timing of the first spike. The maximum membrane potential after the first spike, $\bar{t}_{\max}$, is given by the solution to

$$E(\bar{t}_{\max})=\int_{t^{(\alpha)}-t^{(\alpha-1)}}^{\bar{t}_{\max}}\frac{dt'}{\tau}e^{-\frac{(\bar{t}_{\max}-t')}{\tau}}E(t'). \qquad \text{(SI.36)}$$

The lower bound on the maximum weight is when the membrane potential reaches spiking threshold at $t=\bar{t}_{\max}$:

$$W_{\alpha,\max}=\frac{V_{th}-V_{rest}}{\int_{t^{(\alpha)}(W_{\alpha,\max})-t^{(\alpha-1)}}^{\bar{t}_{\max}}\frac{dt'}{\tau}e^{-(\bar{t}_{\max}-t')/\tau}E(t')}. \qquad \text{(SI.37)}$$

Note that $t^{(\alpha)}$ is a function of W_α and therefore this equation is an implicit equation.

A refractory period will increase the lower bound on maximum synaptic weight. To see this, note that the maximum membrane potential after the first spike, $\bar{t}_{\max}$, is now given by the solution to

$$E(\bar{t}_{\max})=\int_{t^{(\alpha)}-t^{(\alpha-1)}+t_r}^{\bar{t}_{\max}}\frac{dt'}{\tau}e^{-\frac{(\bar{t}_{\max}-t')}{\tau}}E(t'), \qquad \text{(SI.38)}$$

where t_r is the refractory period. The lower bound on the maximum weight is when the membrane potential reaches spiking threshold at $t=\bar{t}_{\max}$:

$$W_{\alpha,\max}=\frac{V_{th}-V_{rest}}{\int_{t^{(\alpha)}(W_{\alpha,\max})-t^{(\alpha-1)}+t_r}^{\bar{t}_{\max}}\frac{dt'}{\tau}e^{-(\bar{t}_{\max}-t')/\tau}E(t')}. \qquad \text{(SI.39)}$$

Taking a derivative of this results with respect to t_r results in a positive number, proving our claim that refractory period increases the lower bound on maximum synaptic weight.

Supplementary Figure 1:

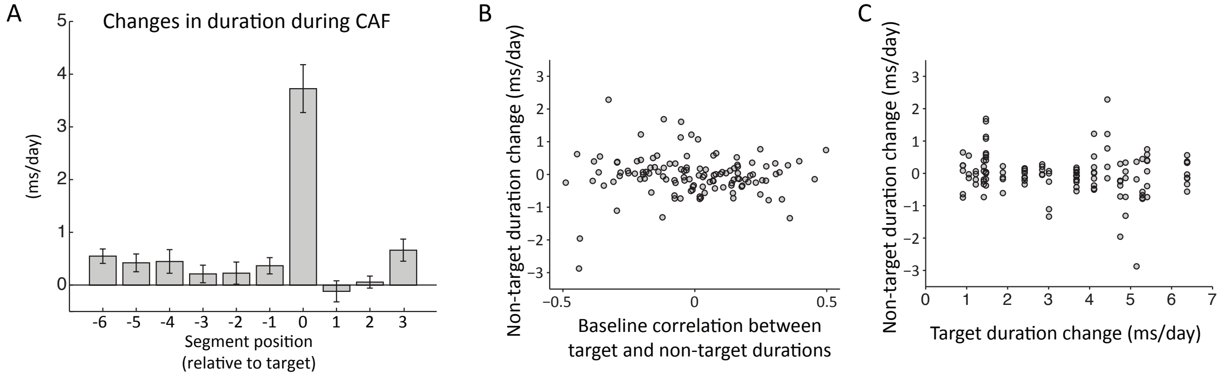

Supplementary Figure 1: Specificity is not related to baseline timing correlations

(A) Changes (non-baseline subtracted) in song segments relative to the targeted segment. **(B)** We analyzed in greater detail how non-target changes (interference) depended on 1) the baseline timing correlation between target and non-target (x1) or 2) the extent of target changes (x2) or 3) both. In all cases, there was no dependence. Non-target changes during CAF did not tend to be larger when the pre-CAF baseline timing correlation between target and non-target interval durations was higher (Pearson's r = -0.008, p = 0.93). **(C)** Non-target changes during CAF were also not larger when the targets were modified to a greater extent (Pearson's r = -0.14, p = 0.11). To test whether both the baseline timing correlations and target changes contribute to non-target changes (i.e., non-targets that have the highest correlation with the target when the target was modified the most might exhibit most interference), we used multiple regression with three explanatory variables: x1 and x2 as stated above and an additional interaction term (product of the x1 and x2 since they are continuous variables). We found no correlation (R^2 = 0.052, p = 0.10). Overall, detailed analysis strongly suggests specificity in birdsong timing, i.e., modifications to one part of a sequence leaves the temporal structure of other parts unaffected regardless of any baseline timing correlations.

Supplementary Figure 2:

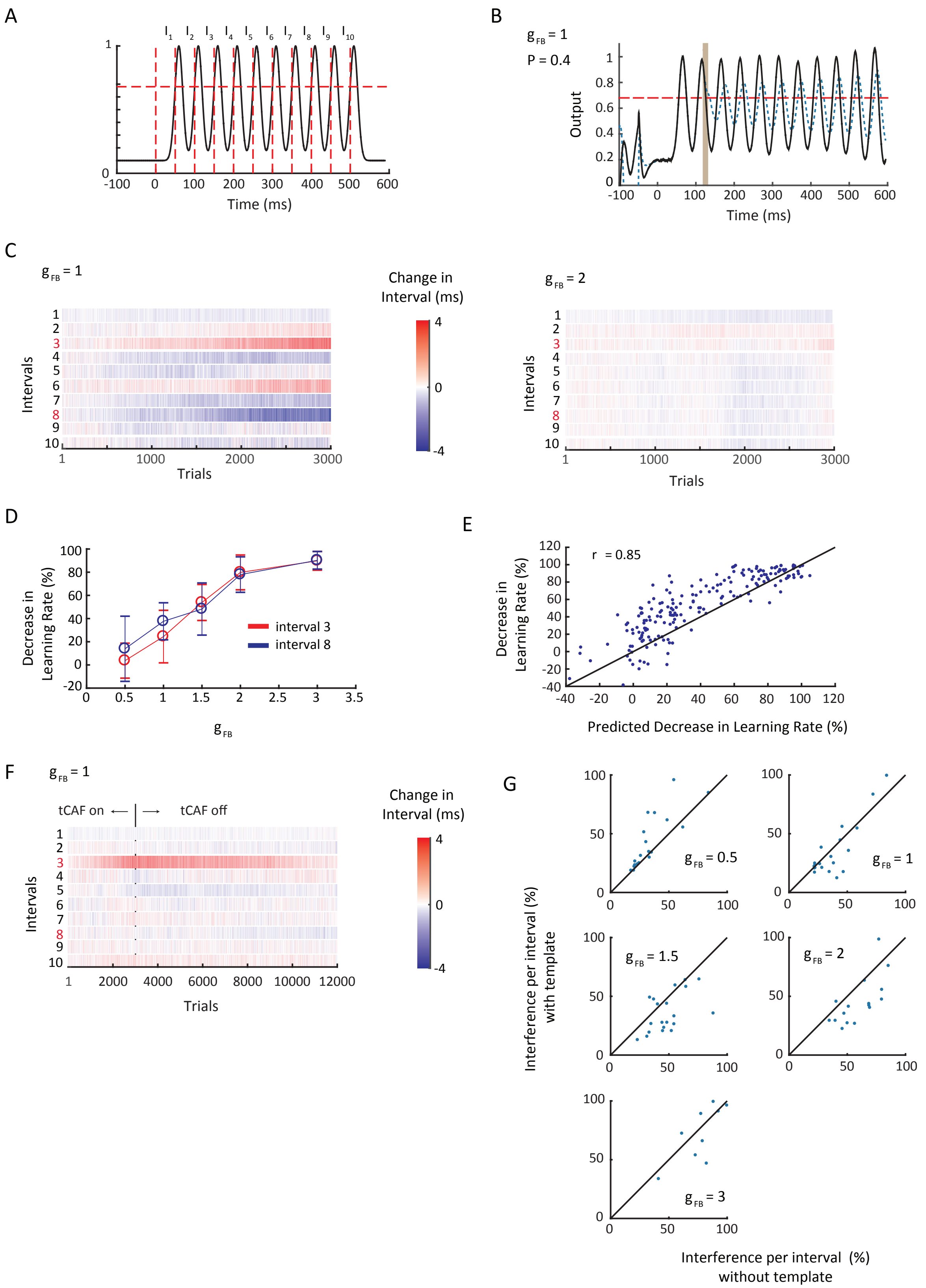

Supplementary Figure 2: Flexibility and robustness of fsRNNs

(A) The signal that the fsRNN and the dynamic attractor networks were trained to reproduce. Interval boundaries (vertical red dotted lines) defined by the signal crossing a threshold (horizontal red dotted line). **(B)** An example of what happens when the network is perturbed, and fails to produce the right timing interval. The black line shows a successful output when no perturbation is delivered. Dashed blue line shows the output when perturbation was delivered to the network during the time denoted by the brown bar. Even though the output crossed the threshold sufficiently many times, the interval durations were not within the desired 6% of their targets. **(C)** Example reinforcement learning simulations for a two-target 'experiment' run for 3000 trials in a fsRNN, where the 3rd and 8th interval was targeted for lengthening and shortening respectively. Shown for different feedback strengths. **(D)** Decrease in average learning rates of the 3rd and 8th intervals (across 20 simulations) when they were targeted together relative to when they were targeted alone. For each network, average learning rates (across 20 simulations) were calculated (as for Figure 2J) for both intervals in single-target and two-target 'experiments'. Error bars are standard deviations across 20 networks. **(E)** Reduction in learning rate is well predicted by the interference matrices. Each dot represents a network-interval pair, pooled over 100 trained fsRNNs (20 for each of g_{FB}=0.5,1,1.5,2,3) and 2 intervals each (3rd and 8th). Reduction in average learning rate (across 20 simulations) plotted against $\frac{M_{\beta\alpha}}{M_{\alpha\alpha}} \times 100\%$ (see Eq. 4 of main text) for that network. (Pearson's r=0.85) **(F)** Example reinforcement learning simulations for a single-target (3rd interval) 'experiment' with template reinforcers for all intervals. After 3000 trials CAF was turned off and the template reward restored the baseline interval. **(G)** Scatter plots of interference per interval with and without the template reinforcers, plotted for separate feedback strengths. Each data point represents a trained network.

Supplementary Figure 3:

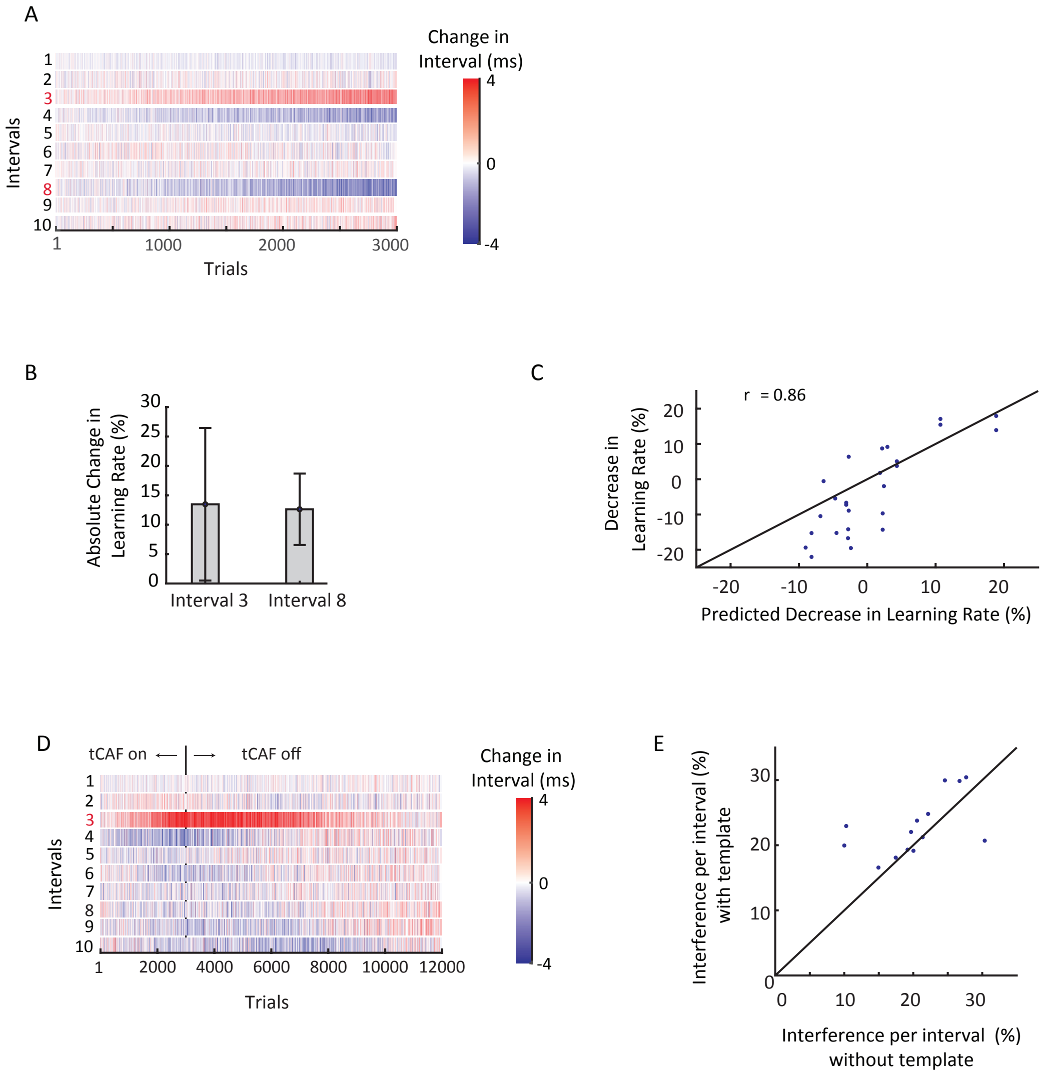

Supplementary Figure 3: Various reinforcement learning simulations in a dynamic attractor
(A) Example reinforcement learning simulation for a two-target 'experiments' run for 3000 trials in a dynamic attractor network, where the 3rd and 8th interval was targeted for lengthening and shortening respectively. **(B)** Absolute change in average learning rates of the 3rd and 8th intervals (across 20 simulations) when they were targeted together relative to when they were targeted alone. Error bars are standard deviations across 14 networks. **(C)** Reduction in learning rate is well predicted by the interference matrices. Each dot represents a network-interval pair, pooled over 14 trained networks and 2 intervals each (3rd and 8th). Reduction in average learning rate (across 20 simulations) plotted against $\frac{M_{\beta\alpha}}{M_{\alpha\alpha}} \times 100\%$ (see Eq. 4 of main text) for that network. (Pearson's r=0.86) **(D)** Example reinforcement learning simulations for a single-target (3rd interval) 'experiment' with template reinforcers for all intervals. After 3000 trials CAF was turned off and the template reinforcement restored the baseline interval. **(E)** Scatter plot of interference per interval with and without the template reinforcers. Each data point represents a trained network.

Supplementary Figure 4:

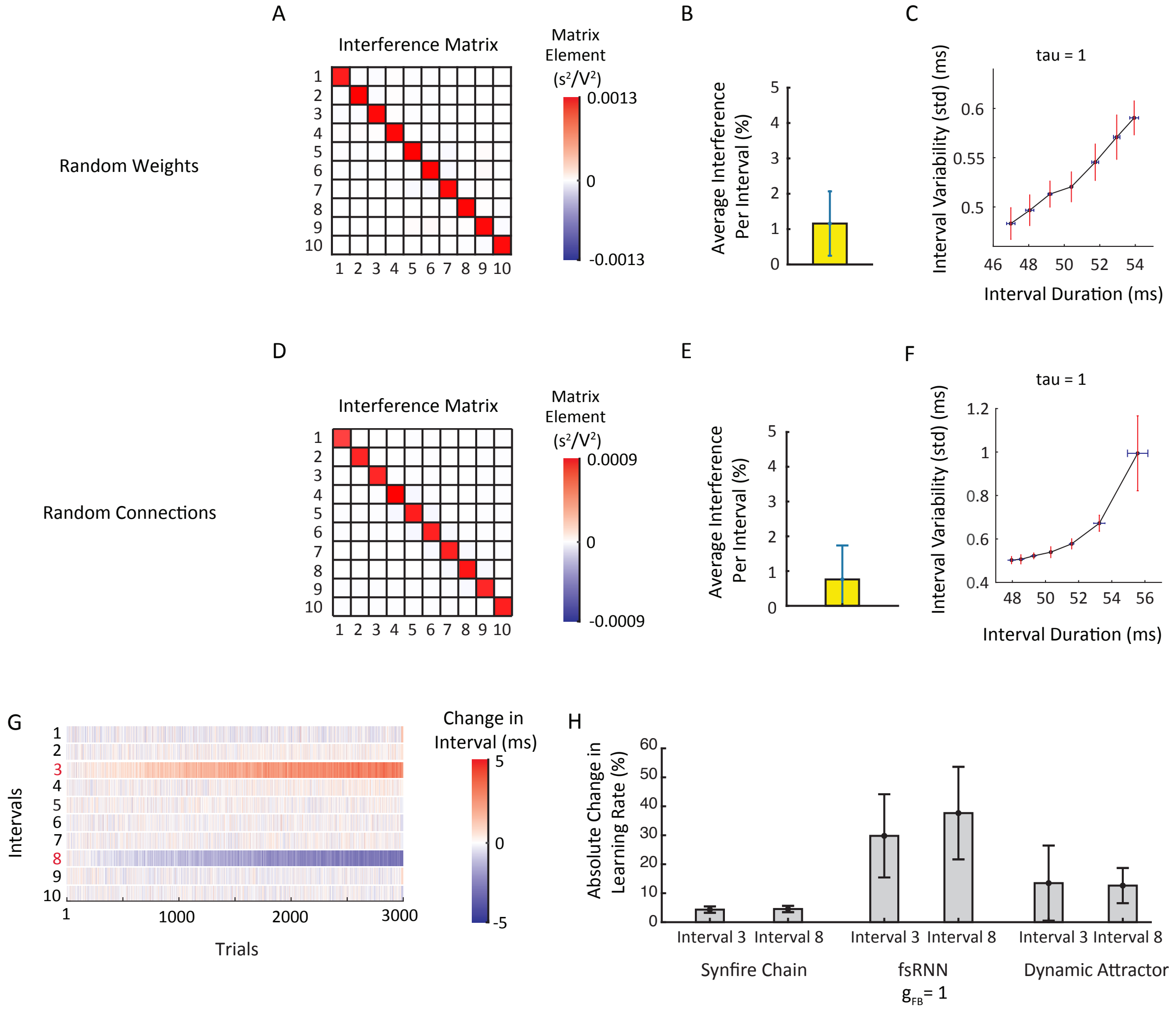

Supplementary Figure 4: Variations of synfire chain architecture and two-target reinforcement learning

(A) Interference matrix for a synfire chain with all-to-all layer-to-layer connectivity and randomly chosen weights. **(B)** Average interference per interval in reinforcement learning simulations, averaged across 20 learning simulations, for the network in (A). Error bar shows standard deviation across simulations. **(C)** Standard deviation of target duration as a function of the target's mean duration, for the network in (A). Data from 200 'catch' trials interspersed between every 1000 trials, averaged across 20 simulations. Target interval was the 3rd. **(D), (E)** and **(F)**, same as (A), (B) and (C) for a synfire chain where neurons make synapses to next layer neurons with probability 0.8. **(G)** Example reinforcement learning 'experiment' for two-target 'experiments' run for 5000 trials in a synfire chain. 3th interval is lengthened and the 8th interval is shortened. **(H)** Absolute change in average learning rates of the 3th and 8th intervals (across 30 simulations) when they are targeted together relative to when they are targeted alone. Error bars are standard deviations across 30 simulations. For comparison, learning rate changes for the fsRNNs and the dynamic attractor are also provided.

Supplementary References:

1. Fiete, I. & Seung, H. S. Gradient learning in spiking neural networks by dynamic perturbation of conductances. *Phys. Rev. Lett.* **97,** 1–4 (2006).

2. Williams, R. Simple Statistical Gradient-Following Algorithms for Connectionist Reinforcement Learning. *Mach. Learn.* (1992).

3. Doya, K. & Sejnowski, T. A novel reinforcement model of birdsong vocalization learning. *Adv. Neural Inf. Process. Syst.* **7,** 101–108 (1995).
